# Narrative coherence of autobiographical memories in women with borderline personality disorder and associations with childhood adversity

**DOI:** 10.1186/s40479-021-00159-5

**Published:** 2021-06-07

**Authors:** Glenn Bendstrup, Erik Simonsen, Mickey T. Kongerslev, Mie S. Jørgensen, Lea S. Petersen, Marianne S. Thomsen, Martin Vestergaard

**Affiliations:** 1 Psychiatric Research Unit, Psychiatry Region Zealand, Slagelse, Denmark; 2 Department of Clinical Medicine, Faculty of Health and Medical Sciences, University of Copenhagen, Copenhagen, Denmark; 3 Psychiatric Clinic Roskilde, Psychiatry Region Zealand, Roskilde, Denmark; 4 Department of Psychology, University of Southern Denmark, Odense, Denmark

**Keywords:** Borderline personality disorder, Narrative coherence, Autobiographical memory, Childhood trauma

## Abstract

**Background:**

People suffering from Borderline Personality Disorder (BPD) seem to have incoherent autobiographical narratives. Tentative evidence suggests that reduced narrative coherence of autobiographical memories is associated with insecure attachment. However, it remains unknown whether incoherent autobiographical narratives in people with BPD are coupled to experiences of childhood trauma, which is highly prevalent in BPD.

**Method:**

We examined if written autobiographical memories in 26 female participants with BPD had reduced narrative coherence relative to 28 healthy female controls and whether more incoherent narratives were associated with childhood trauma.

**Results:**

As hypothesized, results showed that compared to controls, the autobiographical memories in participants with BPD had reduced narrative coherence, specifically inadequate orientation about the narrative and lack of narrative structure. More self-reported childhood adversity was coupled to lower orientation across groups whereas increased childhood adversity showed a specific relationship to lowered narrative structure in BPD participants.

**Conclusion:**

Women with BPD had incoherent autobiographical narratives, and reduced narrative coherence was associated with more self-reported childhood adversity, which appeared to explain the group differences.

## Introduction

Autobiographical memories are the subjective recollections of experienced events that make up the building blocks of our personal life-narratives [1, 2]. There is wide agreement that self-guiding, socially-oriented and directive functional properties of autobiographical memories are central to our mental health [3]. Autobiographical memories provide us with a sense of having a continuous self across time and space [4], and have a social function as we share our personal experiences with each other to nurture and facilitate social relationships [5]. Autobiographical memories also have a directive function and guide our problem-solving and goal-oriented behavior [6, 7]. Given that the core symptoms in borderline personality disorder (BPD) include a disturbed sense of self, unstable social relationships and affective and behavioral dysregulation [8], it seems probable that the functional properties of autobiographical memories are compromised in people with BPD.

### Narrative coherence in borderline personality disorder

Autobiographical memories are remembered and communicated as narratives. The competence to remember autobiographical memories as coherent narratives provides us with a temporal and causal framework that integrates past and current experiences into a unified and evolving life-story, thereby making sense of an otherwise myriad of unconnected events that we as individuals experience [9]. Better autobiographical narrative coherence shows a positive relationship with psychological well-being [4, 9–11].

The ability to convey episodic memories as coherent narratives develops throughout childhood and adolescence [12], and studies have shown that when mothers elaborate more while reminiscing about past events together with the child, those children generate more coherent narratives [13], have a more consistent self-concept [14], display more secure attachments and have better impulse control [15]. Recently, a study in psychiatric inpatient adolescents showed that more coherently narrated episodic memories were correlated with higher self-reported attachment security and less externalizing behavior [16]. A longitudinal intervention study with focus on attachment style in people suffering from BPD showed that following 1 year of treatment with transference-focused psychotherapy, the BPD participants’ narrative coherence on the Adult Attachment Interview (AAI) increased significantly, which correlated with increases in self-reported reflective functioning and a more secure attachment style [17]. Prevalence rates of self-reported childhood trauma are exceedingly high in BPD, with a recent meta-analysis estimating that individuals with BPD are 13.9 times more likely to report childhood adversity relative to healthy controls [18]. Because insecure attachment and traumatic experiences in childhood are considered to increase the risk of developing BPD later in life [19, 20], self-reported experiences of abuse and neglect are perhaps coupled to more incoherent autobiographical narratives in adulthood.

More borderline-related traits, in otherwise healthy individuals, have been associated with worse narrative coherence [21]. Jørgensen et al. [22] used a categorical procedure to code the narrative style of autobiographical memories and showed that, compared to healthy controls, the autobiographical memories in participants with BPD were more frequently disoriented, defined as a narrative that jumps incoherently from one event to another, leaving out central parts of the narratives. Furthermore, Jørgensen et al. [22] observed that the autobiographical memories in individuals with BPD or Obsessive-Compulsive Disorder (OCD) were more frequently impoverished relative to controls, defined as a narrative that consisted of too few sentences to be meaningfully analyzed [22]. Another study used the same categorical procedure to assess narrative coherence and reported that compared to healthy controls, the narratives in BPD outpatients were more impoverished [23]. Thus, the few studies published to date indicate that individuals with BPD narrate their autobiographical memories less coherently than healthy controls [22, 23]. However, it is unknown whether incoherent autobiographical narratives in BPD are related to experienced childhood adversity, which is a key characteristic of the disorder.

### Episodic specificity in borderline personality disorder

The functional properties of autobiographical memories are not solely dependent on narrative coherence but also whether the individual is able to retrieve specific episodic memories rather than more generalized personal memories [2, 3]. Trouble retrieving specific episodes from one’s past has been linked to increased self-reported experiences of childhood and adult psychological trauma [24, 25] and depression [26]. While some studies have shown autobiographical memories to be less specific in individuals with BPD compared to healthy controls [23, 27–31], others have reported no such differences [22, 32–34]. One study reported that apparent differences in episodic specificity of autobiographical memories between BPD subjects and controls were explained by differences in intelligence and educational level [30]. Although it is uncertain why findings on episodic specificity in BPD are mixed, episodic specificity in individuals with BPD may vary dependent on whether the autobiographical memories recalled, primarily have a social, self-relevant or directive functional property.

### The current study

We hypothesized that women diagnosed with BPD would convey autobiographical memories less coherently than healthy women. Follow-up analyses were performed to examine whether incoherent autobiographical narratives were related to more self-reported childhood trauma in participants with BPD and controls, as well as with borderline symptoms in the BPD group. The validated coding scheme for narrative coherence developed by Baerger & McAdams [9] was employed to analyze written autobiographical memories. We decided to use this coding scheme as the output from the Baerger & McAdams’ is scored on a dimensional scale and is therefore more fit for linear analyses than categorical coding schemes [22, 23]. We further explored whether the autobiographical memories of BPD participants were less specific than controls, and whether BPD participants showed decreased narrative coherence and episodic specificity for the self, social and directive functions of autobiographical memory, respectively.

## Method

### Participants

The present study examined narrative coherence of autobiographical memories in a subsample of female BPD participants and healthy female controls from a larger cohort reported elsewhere [35]. Twenty-six women aged 18–45 years who met diagnostic criteria for BPD according the Diagnostic and Statistical Manual of Mental Disorders, Fourth Edition (DSM-IV) were included in the study. Twenty-eight controls were matched on age and sex, as well as parental education to assure that BPD participants and controls did not differ in socioeconomic status of the family of origin (missing data on parental education for two BPD participants). Parent education was coded in one of five categories (1 = no education; 2 = trained worker; 3 = skilled worker; 4 = theoretical education of lower academic level; 5 = academic education). When data on parental education was available for both parents, the mean of the parental education categories was reported. We furthermore recorded the educational status for each subject to control for inter-individual variability in educational level coded in one of six categories (1 = no education or primary school; 2 = skilled worker; 3 = university-preparatory school; 4 = short academic education; 5 = academic education equivalent to a bachelor’s degree; 6 = academic education equivalent to a master’s degree), one BPD participant had missing data on subject education. The matching and control variables are displayed in Table 1.

**Table 1:** Matching variables, control measures, symptom scale, and psychopathology scales for patients and controls

	BPD subjects (*N* = 26)	Controls (*N* = 28)
**Control variables**		
Age	28.67 ± 7.21	28.88 ± 8.77
Parental education	2.45 ± 0.96	2.71 ± 0.79
Subject education	2.52 ± 1.68	3.85 ± 1.32
WAIS VCI	91.15 ± 10.4	103.32 ± 6.2
Memory word count	75.04 ± 34.9	84.63 ± 29.3
CTQ	60 (44–71)	28 (25–33)
**Symptom scale**		
ZAN-BPD	12.6 ± 6.4	–
**Psychiatric diagnostic comorbidity** ***n*** **(%)**		
Major Depressive Disorder	3 (11.5)	0 (0.0)
Bipolar II Disorder	0 (0.0)	0 (0.0)
Agoraphobia	6 (23.1)	0 (0.0)
Panic Disorder without Agoraphobia	7 (26.9)	0 (0.0)
Social Phobia	7 (26.9)	0 (0.0)
General Anxiety Disorder	2 (7.7)	0 (0.0)
Obsessive-Compulsive Disorder	2 (7.7)	0 (0.0)
Posttraumatic Stress Disorder	6 (23.1)	0 (0.0)
**Personality disorders** ***n*** **(%)**		
Avoidant	9 (34.6)	0 (0.0)
Dependent	2 (7.7)	0 (0.0)
Obsessive-Compulsive	6 (23.1)	0 (0.0)
Paranoid	5 (19.2)	0 (0.0)
Schizotypal	0 (0.0)	0 (0.0)
Schizoid	0 (0.0)	0 (0.0)
Histrionic	2 (7.7)	0 (0.0)
Narcissistic	2 (7.7)	0 (0.0)
Antisocial	2 (7.7)	0 (0.0)

Data is shown with mean ± standard deviations except the CTQ score, which is displayed with medians and lower and upper quartiles because the variable significantly deviated from the normal distribution. Missing data is described in the method section. *WAIS VCI* Wechsler Adult Intelligence Scale Verbal Comprehension Index, *CTQ* Childhood Trauma Questionnaire, *ZAN-BPD* Zanarini Rating Scale for Borderline Personality Disorder

Exclusion criteria for all participants included DSM-IV lifetime psychotic disorder, bipolar I disorder or substance use disorder, history of significant head trauma, severe chronic physical or neurological illness such as seizure disorder, encephalitis or stroke or a VCI score below 70 points.

BPD participants were recruited from Psychiatric Clinic Roskilde, an outpatient clinic in Region Zealand Psychiatry, Denmark, specialized in treating BPD, and controls were recruited through local advertisement. All participants received thorough written and verbal information about the project and were required to provide written informed consent before inclusion in the study. The study was approved by the Regional Ethics Committee for Science Ethics of Zealand. Recruitment procedures are described in more detail elsewhere [35].

### Assessments

#### Diagnostic assessment

BPD participants and controls were screened for psychiatric disorders by trained clinicians with the Mini International Neuropsychiatric Interview [36], and for personality disorders with the Structured Clinical Interview for DSM-IV Axis II Disorders [37]. All subjects in the BPD cohort fulfilled the criteria of BPD.

#### Borderline symptoms

Severity of borderline symptoms within the last 2 weeks was assessed in BPD participants with the nine-item Zanarini Rating Scale for Borderline Personality Disorder (ZAN-BPD), which is a clinician-administered semi-structured interview [38]. The items, each of which reflect the core symptoms associated with BPD, were scored on a scale ranging from 0 to 4 based on frequency and severity of BPD symptoms. The ZAN-BPD total score is presented in Table 1.

#### Childhood trauma

We assessed childhood trauma experiences with the validated Danish version of the Childhood Trauma Questionnaire (CTQ) [39]. The CTQ is a standardized retrospective 28-item self-report inventory that measures the occurrence and severity of experienced childhood trauma including emotional, physical and sexual abuse, emotional and physical neglect scored on a 5-point Likert scale [40]. We used the total score in the present study. Three BPD participants and one person in the control group had missing data on the CTQ.

#### Verbal comprehension index

We have previously shown that the BPD participants in the present study displayed lower verbal comprehension relative to controls [35, 41] and the VCI scale has been shown to be positively associated with reading comprehension and spelling [42]. Because our participants were asked to write down their autobiographical memories, individual differences in verbal comprehension abilities could affect how the autobiographical memories was communicated on paper. Therefore, we assessed individual differences in verbal knowledge and reasoning with the Similarities, Vocabulary and Information subscales from the Wechsler Adult Intelligence Scale, Fourth edition [43], which were combined to estimate the Verbal Comprehension Index (VCI).

### Autobiographical memory

Participants were asked to recall autobiographical memories of specific events from their lives. Each participant was asked to recall a total of six autobiographical memories, two of which had to be self-guiding, socially shared, and directive in theme, respectively. The participant was requested to recall either a self-guiding, socially shared or directive autobiographical memory by cue-card with one of the following prompts: *Try recalling an event that you think says something about your identity* (Self-guiding autobiographical memory), *Try recalling an event that you often share with other people* (Socially shared autobiographical memory), and *Try recalling an event that you think of in order to solve current or future problems* (Directive autobiographical memory).

Participants were presented with one cue-card at a time in a randomized order. The response time was recorded from when a cue-card was shown until the participant verbally indicated that a memory had been recalled. If the participant had not responded after three minutes, the trial was registered as an omission. Participants were instructed to write down the memories on paper with no time restraint and were instructed to communicate each event as if they told it for the first time to a new friend with whom they felt comfortable. Eight BPD participants had a total of 15 omissions, including 6 directive, 5 social and 4 self-guided memories, while we registered a total of 4 omissions across four controls including 3 directive memories and 1 social memory.

Narrative coherence was coded using an adapted version of the coding system developed by Baerger & McAdams (1999), which has previously been used in adult [21] and adolescent [44] clinical samples. Narrative coherence was operationalized using four subscales: Orientation, Structure, Affect and Integration. To ensure the reliability of the scoring procedure, we simplified the original scale, which ranged from 1 to 7 points [9], to a 5-point scale with 1 representing minimum coherence, and 5 maximum coherence for each of the four subscales. A similar simplified adaption of the coding procedure has been successfully employed before [21, 44]. A global score for total narrative coherence was calculated based on the average of the four subscales. The Orientation subscale measures how satisfactorily the story provides the reader with adequate context to comprehend the story. Context includes critical background information leading up to the beginning of the story, including sufficient information about the main characters, and when and where the story takes place. The Structure subscale evaluates the logical flow of the story and whether events and reactions appear causally and temporally connected. A story scoring high in structure should also display the structural elements of an episode system described in detail elsewhere [9]. The Affect subscale measures how coherently affective words or phrasings are incorporated to make evaluative points, giving a clear indication of the feelings experienced by the persons described in the autobiographical memories. The Integration subscale pertains to how skillfully the narrator communicates the point of telling the story. Autobiographical memories high in integration convey the significance of the experience to the narrator and how it relates to the narrators’ overall life. Descriptive statistics for narrative coherence scores are displayed in Table 2.

**Table 2:** Descriptive statistics for outcome measures for the patient and control group

	BPD subjects (*N =* 26)	Controls (*N =* 28)
**Outcome measures**		
Episodic specificity	1.02 ± 0.75	1.11 ± 0.73
Orientation subscale	2.64 ± 0.69	3.23 ± 0.70
Structure subscale	2.71 ± 0.72	3.35 ± 0.74
Affect subscale	2.30 ± 0.70	2.18 ± 0.55
Integration subscale	2.37 ± 0.57	2.72 ± 0.56

Data is shown with group mean ± standard deviations. The displayed data for the narrative subscales and episodic specificity score is based on each subject’s mean scores from the six autobiographical memories

Memories were considered specific if they were situated in a specific time and place, typically not exceeding the course of a day. Episodic specificity was scored on a scale ranging from 0 to 3. A score of 0 points indicated a generalized memory with no episodic details, a 1-point score indicated an autobiographical memory with few and fragmented episodic details, a memory that scored 2 points included some references to specific and associated episodic details, and a memory that scored 3 points was characterized by being rich in episodic details, and firmly situated in time and place. The episodic specificity score is presented in Table 2.

#### Inter-rater reliability

Three blinded coders (G.B, M.S.J and L.S.P) rated the Episodic specificity score, and the Orientation, Structure, Emotion and Integration subscale scores, respectively, based on anonymized data from 30 randomly selected participants. Inter-rater reliability was estimated from the mean score of the six autobiographical memories for each participant for the Episodic specificity, Orientation, Structure, Emotion and Integration scores, respectively. Inter-rater reliability was estimated using intraclass correlation coefficient (ICC) using a two-way random model with absolute agreement for single measures. Results showed a moderate to good reliability for the Episodic specificity score (ICC = 0.880), and the Orientation (ICC = 0.655), Structure (ICC = 0.766), Emotion (ICC = 0.703) and Integration (ICC = 0.785) subscale scores. Because the inter-rater reliability was satisfactory, we used the first author’s ratings for the statistical analyses of all participants.

### Statistical analysis

Statistical analyses were conducted in SPSS 25. A *p* value below .05 was considered significant. Group differences for the matching and control variables were tested with two-tailed *t* tests. Group differences in omissions were tested with the non-parametric Mann-Whitney U test.

To assure that group differences were not explained by potential confounders, all ANCOVA and multiple linear regression models were controlled for age, subject education, parent education, VCI score and number of words used in the autobiographical stories. We used a two-way repeated measures ANCOVA to test our primary hypothesis that the autobiographical memories in BPD participants had lower narrative coherence compared to controls and to test for group differences in the social, self and directive memory functions. Group was entered as the between-subject factor while the narrative subscales were entered as the first within-subject factor and the memory functions were entered as the second within-subject factor in the ANCOVA model. To test group differences in episodic specificity, we likewise used repeated measures ANCOVA, in which group was entered as the between-subject factor while episodic specificity for the social, self and directive scores were entered as within-subject factor. If significant results were observed, multiple linear regression were used to test the directionality of the observed group differences. Planned follow-up analyses were performed to examine if significant group differences were coupled to self-reported childhood adversity. Initially, the CTQ score was entered as a control variable to see if group differences persisted when controlling for childhood trauma. Subsequently, we included an interaction term for group by CTQ score to examine if the respective outcome measure showed a different relationship with experienced childhood adversity in BPD participants relative to controls. If the interaction term was not significant, we explored whether the CTQ score was associated with the outcome measure across BPD participants and controls. Lastly, we planned to explore whether the outcome measure was associated with symptom severity in BPD participants entering the ZAN-BPD score as the predictor of interest. For the covariates used in the multiple linear regression models, missing values were replaced by the mean of the respective variable to limit unwarranted exclusion of subjects. All multiple linear regression models were visually inspected to ensure normal distribution of the residuals and all model covariates fulfilled criteria of noncollinearity with Tolerance >.3.

Continuous variables were determined to have a significantly non-normal distribution if either of the respective standard errors for the skewness or kurtosis were above or below Z ± 1.96 (two-sided *p* < .05) in the BPD participants and/or controls. Non-normally distributed variables were normalized using the Rankit transformation, and the normalized values were used in the statistical analyses. The CTQ score was significantly non-normally distributed (*p <* .05) and was successfully normalized with Rankit transformation. The remaining variables all appeared normally distributed.

## Results

BPD participants and controls were matched on age (t (52)=0.092; *p* = .927) and parental education (t (50)=1.046; *p* = .300). As previously reported in a larger sample, the BPD participants had a significantly lower educational level (t (51)=3.227; *p* = .0002), poorer VCI score (t (52)=5.265; *p* < .0001), and had more self-reported experiences of childhood trauma on the CTQ (t (48)= − 7.502; *p <* .0001) relative to controls [35, 41]. No significant difference between BPD participants and controls was observed in the number of words used to narrate their memories (t (52)=1.096; *p* = .278). The Mann-Whitney U test showed no significant group differences in total omissions (U = 434; *p* = .095). Bivariate correlations between the narrative coherence subscales and episodic specificity across BPD subjects and controls are displayed in Table 3.

**Table 3:** Bivariate correlations between the narrative coherence subscales and episodic specificity across BPD subjects and controls

	1	2	3	4
1. Orientation subscale				
2. Structure subscale	.897^**^			
3. Affect subscale	.486^**^	.522^**^		
4. Integration subscale	.549^**^	.552^**^	.490^**^	
5. Episodic specificity	.459^**^	.592^**^	.313^*^	−.086

^*^ *p* < .05

^**^ *p* < .01

### Group differences in narrative coherence

No main effect of group was observed on the repeated measures ANCOVA for the average of the subscales F (1, 47)=2.004; *p* = .16). We did, however, see a significant group by narrative subscale interaction (F (3, 141) = 4.060; *p* = .008), but not a group by memory function interaction (F (2, 94) = .004; *p* = .99), or a group by narrative subscale by memory function (F (6, 282) = 1.172; *p* = .32). Multiple linear regression analyses based on each subject’s mean scores from the six autobiographical memories showed that relative to controls, BPD participants scored lower on the Orientation subscale (t (47)= − 2.266; β = −.287; *p* = .028) and Structure subscale (t (47)= − 2.153; β = −.219; *p* = .036). No apparent group differences were observed for the Affect subscale (t (47)=1.441; β = .205; *p* = .16) or for the Integration subscale (t (47)= − 1.005; β = −.158; *p* = .32).

### Group differences in episodic specificity

The repeated measures ANOVA showed no main effect of group for the average episodic specificity (F (1, 47)=.929; *p* = .34) or a significant interaction for group by autobiographical memory function for episodic specificity (F (2, 94) = 1.863; *p* = .16).

### Follow-up analyses

**Fig. 1:**
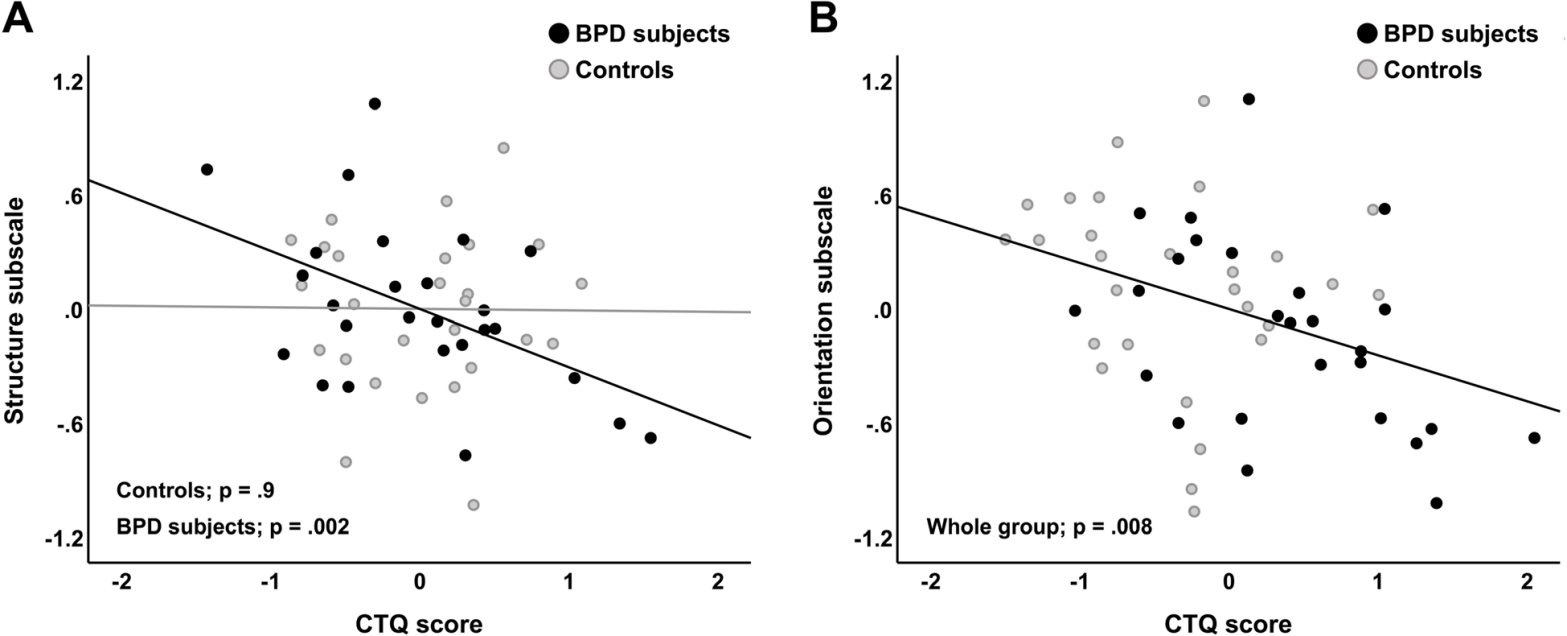
The partial regression plots display whole-group association between the CTQ score and the Orientation subscale across patients and controls (**A**), and the significant interaction effect for group by CTQ score with the Structure subscale (**B**) as illustrated by the separate regression lines for patients and controls. The patients are coded in black and controls are coded in grey. The values displayed on the Y-axes and X-axes are the regression model residuals. The plots are corrected for age, parent education, subject education, the VCI score and word count

When the significant group differences in Orientation and Structure were corrected with the CTQ score, the group differences did not persist neither for the Orientation (t (46)= − .794; β = −.125; *p* = .43) or the Structure (t (46)= − .551; β = −.069; *p* = .58) subscale. However, we observed a significant group by CTQ interaction for the Structure subscale (t (45)= − 2.110; β = −.156; *p* = .040; Fig. 1A), whereas no group by CTQ interaction was seen for the Orientation subscale (t (45)= − .674; β = −.065; *p* = .50). Multiple linear regression within-group analyses showed that the CTQ score was negatively related to the Structure subscale in BPD participants (t (19)= − 3.564; β = −.368; *p* = .002) while a significant relationship between the Structure subscale and the CTQ score appeared absent in controls (t (21)=.111; β = .014; *p* = .91). Whole-group analyses across BPD participants and controls for the Orientation subscale showed a negative association with the CTQ score (t (47)= − 2.752; β = −.293; *p* = .008; Fig. 1B). In BPD participants, the ZAN-BPD was not significantly associated with the Orientation (t (19)= − .146; β = −.026; *p* = .89) or Structure (t (19)= − .747; β = −.114; *p* = .46) subscale.

## Discussion

As hypothesized, women with BPD showed reduced narrative coherence in their autobiographical memories compared to healthy controls. Specifically, incoherent narratives in BPD participants were driven by decreased scores on the Orientation and Structure subscales. That is, BPD participants did not communicate their autobiographical memories with sufficient context leading up to the story and their narratives lacked a logical and causal flow compared to controls. We found no apparent group differences on the Affect and Integration subscale, which indicate that BPD participants and controls use affect similarly to narrate their memories and communicate the points of their memories equally well. Our findings are in agreement with earlier studies, which showed that relative to healthy controls, autobiographical narratives in BPD individuals were more disoriented and impoverished [22, 23], corresponding to a lower score on the Orientation and Structure subscales in the present study. Our results are also somewhat in agreement with a study reporting that an increased number of BPD symptoms were associated with lowered narrative coherence in undiagnosed adults [21]. However, we did not observe a significant relationship between the Orientation and Structure subscales and the ZAN-BPD score in the BPD subjects, this may be related to a lack of power. The observed group differences in Orientation and Structure could not be explained by differences in age, parental education, subject education, verbal comprehension or number of written words.

### Narrative coherence and associations with childhood trauma

In the present study, self-reported childhood trauma as measured with the CTQ, displayed a negative relationship with the Orientation subscale across BPD participants and controls. Interestingly, the Structure subscale was negatively associated with self-reported experiences of childhood neglect and abuse in women with BPD, but not in controls. Childhood adversity and insecure attachment may impair the ability to make coherent narratives. It has been shown that children exposed to heightened family conflict had more incoherent narratives on the MacArthur Story Stem Battery, which were coupled to more externalizing behavior [45]. Adolescents suffering from predominantly depression and/or anxiety had lowered narrative coherence on the Child Attachment Interview, which was related to more insecure attachment [16], and increased number of borderline symptoms [44]. Moreover, children of mothers with BPD display reduced narrative coherence on story telling [46]. However, the competency to develop coherent narratives in childhood may also act as a compensatory mechanism. Better coherence on the MacArthur Story Stem Battery in preschool children was observed to attenuate the otherwise positive relationship between increased maternal psychological stress and internalizing symptoms in the child [47], as well as how seriously family risk factors affected the manifestation of internalizing symptoms and externalizing behavior [48]. While more childhood trauma may not be specific to the observed lower narrative coherence in BPD, individuals with BPD have a high prevalence of experienced childhood trauma [18], which therefore places them at increased risk of having incoherent autobiographical memories. Traumatic parenting predisposes insecure childhood attachment and in the absence of dyadic parent-child reminiscing presumably undermines the developing child’s capacity to mentalize, form a stable identity and trust others. This ultimately leading to epistemic mistrust and mentalizing deficits, which lies at the heart of BPD [20, 49].

Our finding that incoherent autobiographical narratives in participants with BPD are closely associated with their experiences of childhood trauma indicate that the assessment of narrative coherence in BPD is of significant clinical and therapeutic value. This is substantiated by an earlier study, which observed that transference-focused psychotherapy seemed to increase BPD subjects’ narrative coherence on the AAI [17]. Related findings have been reported by studies in patients diagnosed with PTSD who had better organized trauma-related narratives following psychotherapy [50, 51]. However, a more recent study observed no apparent relationship between the coherence of narrative reflections about getting therapy and improvements in mental health symptoms following psychotherapeutic treatment in a group of adult men and women who were treated at an outpatient mental health clinic for various reasons [52].

It may also be that the lowered orientation and narrative structure in the BPD subjects is partially coupled to reduced mentalizing skills, which is commonly observed in people with BPD [20, 49]. This is in line with evidence that better narrative coherence on the AAI is correlated with more reflective functioning about mental states in BPD subjects [17], and in community adolescents [53]. Because a high score on the Orientation subscale requires the subject to provide the recipient with sufficient information about the context leading up to the story, and the Structure subscale measures the logical flow and whether events appear causally connected, both scales undoubtedly require some level of mentalizing skills. However, this question remains to be examined in future studies.

The group differences in narrative coherence did not seem specific to either the social, self or directive functions of autobiographical memory, which could suggest that the incoherent narratives of autobiographical memories seen in BPD affect the autobiographical functions globally. Moreover, groups did not differ in episodic specificity concurrent with some earlier studies in BPD [22, 32–34] but not others [23, 27–31].

### Study limitations

We instructed participants to recall singular autobiographical events rather than asking them to recall a series of interconnected autobiographical memories that made up their life story as was the original intention with the Baerger and McAdams [9] coding scheme. As we did not require the subjects to connect a sequence of autobiographical events into a meaningful life story, it is likely that the recording of singular autobiographical memories in our study did not adequately tap into the need for using Integration and Affect. This might explain why neither the Affect nor the Integration subscales differed significantly between BPD participants and controls.

We asked subjects to write down their autobiographical memories, which may have placed higher demands on lexical skills compared to oral recall. However, our results are in agreement with Jørgensen et al. [22] who likewise asked subjects to write down their autobiographical memories, as well as with other studies that requested subjects to orally recall autobiographical memories [21, 23], suggesting that narrative coherence is consistently disturbed in BPD, regardless of how autobiographical memories are reported.

Because we did not include other psychiatric diagnostic groups, we are not able to establish whether our findings are specific to the underlying pathology in BPD. In fact, we do not expect that deficits in narrative coherence would display diagnostic specificity to BPD as increased experiences of childhood trauma appeared to explain the group differences in narrative coherence. Furthermore, because of our small sample size we lacked the statistical power to explore if psychiatric comorbidity had an effect on the group differences. That we did not find any significant relation between ZAN-BPD and narrative coherence in patients, may also be related to a lack of power or low variance in the patient sample. Because we did not assess control participants for subthreshold BPD symptoms e.g. with ZAN-BPD we could not explore whether BPD symptoms might have explained the correlation between the coherence subscales and self-reported childhood trauma we observed across the patient and control groups.

Participants were given three minutes to recall an autobiographical memory, and some BPD individuals appeared to have more trouble with recall than controls, although groups did not differ significantly. At group level, however, subjects with BPD had somewhat less autobiographical memory data compared to controls. It is unclear why some BPD participants were not able to recall as many autobiographical memories within the constraint of three minutes. However, studies have associated higher working memory capacity with better and more coherent recall of semantic autobiographical information [54] and more specific autobiographical memories [55] in community adults. As we have previously shown that the BPD subjects in the present study had lower working memory [35], it seems likely that troubles with recall in some BPD individuals are partially related to executive deficits.

Lastly, because our study used a cross-sectional design, our results do not demonstrate causality, but is entirely correlational.

## Conclusion

In conclusion, women with BPD did not provide adequate orientation and narrative structure when narrating their autobiographical memories relative to controls. Notably, we provide novel evidence that incoherent autobiographical narratives seem to be coupled with more experiences of childhood adversity, particularly in women with BPD and to a lesser extent controls. The dynamic nature of narrative coherence might therefore play a critical role in the psychopathology and psychotherapeutic treatment of BPD.

## Data Availability

Not applicable.
